# Association Between Anti‐TIF1‐γ Antibody and Triple‐Negative Breast Carcinoma in Dermatomyositis Patients: A Case Report

**DOI:** 10.1002/ccr3.70157

**Published:** 2025-02-10

**Authors:** Fan Zhang, Hailing Guo, Jia Ma

**Affiliations:** ^1^ Peking Union Medical College Hospital Beijing City China

**Keywords:** anti‐TIF‐γ, breast carcinoma, dermatomyositis, triple‐negative

## Abstract

Dermatomyositis (DM) is always associated with various types of malignancies in previously published reports. Anti‐transcription intermediary factor 1‐γ (anti‐TIF1‐γ) antibody is more prevalent in DM patients with malignancy than in those without malignancy. In this paper, we want to show a possible relation between anti‐TIF1‐γ and triple‐negative breast carcinoma.

## 1 Introduction

With an incidence of approximately 1/100,000, 9.63 per 1,000,000 people, dermatomyositis (DM) is a rare and uncommon idiopathic inflammatory myopathy characterized by varying degrees of skeletal muscle weakness, especially proximal muscle strength, and different well‐spotted cutaneous manifestations [1]. It has been well reported that DM carries a high risk of malignancy and can present as a paraneoplastic syndrome in the circumstance of underlying malignancies. With an estimated 15%–30% of DM patients having an underlying malignancy, a variety of frequencies of malignancy have been found in lung, ovarian, breast, et al. [2]. In between, breast cancer appears to be the most common in established findings [3]. Regarding the relationship between DM and cancer, the treatment of cancer sometimes leads to an improvement in the DM‐related symptoms, especially in the skin presentations and muscle strength [4]. Therefore, it is important to perform enhanced cancer screening and timely treatment for patients with DM [5].

There are a great number of related auto‐immune antibodies, which can be beneficial for diagnosis and prognosis judgment [6]. One (anti‐transcriptional intermediary factor‐1γ, anti‐TIF‐1γ) of typical myositis‐specific autoantibodies has been disclosed recently. It has been recognized that its prevalence in DM patients with malignancy is higher than that in those without malignancy [7]. Hence, it has become a routine test when DM is suspected. However, its underlying association is unclear.

In 2023, 3 cases were diagnosed with breast cancer and DM in our hospital. Because of serious onset of cutaneous presentations, they consulted the dermatology department primarily in other hospitals. Based on the reminder of the dermatologist after treatment without effect, breast carcinoma, and DM were diagnosed via further detailed laboratory and core needle examination. Then anti‐TIF‐1γ positive and trip‐negative breast cancer were identified simultaneously in those 3 patients. There are rare case reports about the incidence of simultaneity after reviewing. Only two similar reports can be found on Pubmed [8, 9], just one case report.

Because of a low incidence of DM and malignancy occurring at the same time, there is no consensus on the treatment priority for DM or malignancy, though it has been proved that treating malignancy first is helpful for DM‐related presentations. Therefore, on one hand, we would like to share our treatment experience in severe DM and malignancy. On the other hand, anti‐TIF‐1γ positive and triple‐negative breast carcinoma co‐exist in four patients. It is imagined whether there is some relationship between them. However, none of the reports mention the possibility. Can we assume that when a DM case with breast cancer is characterized by anti‐TIF‐1γ positive, there will be a high incidence of triple‐negative breast cancer?

## 2 Case History and Examination

Three female patients aged from 41 to 51 years. One of them had a tumor that occurred first and then symptoms of DM were found 2 weeks later, and the rest of the patients were found first with symptoms related to DM and then found with tumors, ranging from 1.5 to 4 months. Skin manifestations in 3 cases: Heliotrope sign, Gottron papules, and Gottron sign in two patients, and Heliotrope sign and Gottron sign in the remaining one patient; other systemic manifestations: all had intermittent dyspnea, choking, and hypoxemia, including 1 case with pulmonary interstitial disease and 1 case with large patchy opacities in the lungs; 1 patient had intermittent dysphagia; one patient had arrhythmia and tachycardia. All 3 patients had muscle weakness, and 2 of them had muscle pain. After the patients were admitted to the hospital with laboratory examination and instrument examination, all patients were positive for antinuclear antibody, anti‐TIF‐1γ positive, combined with skin and muscle weakness. The diagnosis of DM and breast cancer was recognized, and the PET‐CT examination did not show distant metastasis. Because 3 patients were evaluated by rheumatology that the symptoms were severe and could not be treated with surgery or neoadjuvant chemotherapy first, they were recommended to undergo DM treatment, and after about 1 month of therapy, the symptoms of DM were significantly improved. After all laboratory indexes reached generally normal, they were transferred to the department of Breast Surgery for radical breast cancer surgery, postoperative adjuvant chemotherapy, and radiotherapy.

There are 3 cases in this report. The similarities and differences between them are shown in Tables 1, 2, and 3. Written informed consent was obtained from all the patients to publish the article. We selected one typical case who was diagnosed with breast mass first to illustrate in the following parts of “Methods and Conclusion”

**TABLE 1:** Systemic manifestations of dermatomyositis.

Organ system	Manifestations		
	Case 1	Case 2	Case 3
Respiratory	Interstitial lung disease	Intermittent dyspnea	Intermittent dyspnea
			Chocking cough
	Intermittent dyspnea	Patchy shadows on the lungs	Low SPO_2_
	Low SPO_2_	Low SPO_2_	
	Choking cough	Chocking cough	
Gastrointestinal	Intermittent dysphagia	Intermittent dysphagia	None
Cardiac	Arrhythmias	Generally normal	Normal
	Tachycardia		
Muscle weakness	Symmetric proximal muscle weakness in lower and upper extremity	Symmetric proximal muscle weakness in upper extremity and muscle tenderness	Symmetric proximal muscle weakness in waist and muscle tenderness
Skin	Heliotrope sign	Heliotrope sign	Heliotrope sign
	Gottron papules	Gottron papules	Gottron sign
	Gottron sign	Gottron sign	

**TABLE 2:** Four cases of DM in breast cancer patients.

Characteristic	Cases		
	Case 1	Case 2	Case 3
Age (years)	43	41	51
Cancer type	Invasive ductal cancer	Invasive ductal cancer	Invasive ductal cancer
Stage	III	IV	III
Timing of cancer and DM	Cancer occurring first	DM occurring first	DM occurring first
	DM occurring 2 weeks later	Cancer occurring 1.5 weeks later	Cancer occurring 4 weeks later
Receptor status			
ER	Negative	Negative	Negative
PR	Negative	Negative	Negative
HER2	Negative	Negative	Negative
CT scan (Chest)	Interstitial lung disease	Patchy shadows on the lungs	None
PET scan	No metastasis	No metastasis	No metastasis
Antinuclear antibiotics	Positive	Positive	Positive
Anti‐TIF1 γ	Positive	Positive	Positive
Treatment	Methotrexate, immunoglobulin	Methotrexate, immunoglobulin	Methotrexate
	Steroid and modified radical	Steroid and modified radical	Steroid and modified radical
	Mastectomy and chemotherapy and radiotherapy	Mastectomy and chemotherapy and radiotherapy	Mastectomy and chemotherapy and radiotherapy
Recurrence of DM	None	None	None
Recurrence of cancer	None	None	None

**TABLE 3:** Laboratory investigations of CK, LDH in 4 cases (before treatment and after treatment).

Cases	CK(IU/L)		LDH(IU/L)	
	Before	After 1 month	Before	After 1 month
1	1010	146	256	159
2	1435	129	284	143
3	1205	152	293	119

## 3 Methods

In June 2023, a 43‐year‐old female patient who denied any history of smoking, drug abuse, alcohol consumption, and family history of tumors presented with a mass in her right breast but did not seek any medical care. About 2 weeks later, some abnormal skin presentations appeared, including Heliotrope sign, Gottron papules, and Gottron sign: facial edema, V‐sign, diffuse erythema with itching on the trunk, neck, back of hands, and bilateral lateral thighs, dilated telangiectatic erythema, scaling, and lichenification of the skin, et al. Moreover, intermittent dyspnea, choking cough, and dysphagia developed and gradually worsened, accompanied by progressive proximal muscle weakness. She consulted a dermatologist at another hospital, and allergic dermatitis was considered. Within 1 week, the rash covered approximately 60% of her body surface area. However, it was ineffective to use antihistamines. She was treated with 3 days of oral prednisone 50 mg daily and topical corticosteroids for a presumed atopic dermatitis, achieving minimal improvement. Without significant relief, the dermatologist considered tumor‐related DM and reminded the patient of further examination of the right breast mass. Then the patient received a B‐ultrasound examination, showing hypoechoic nodules at the 8‐point, measuring 1.2 × 0.87 × 0.8 cm on the right breast, irregular margins, BI‐RADS 4b. A core needle biopsy was taken to reveal a poorly differentiated invasive carcinoma of the right breast, grade III, and related receptors: estrogen, progesterone receptor, and HER‐2 expression were negative, Ki‐67 (70%+), suggesting triple‐negative breast cancer (TNBC).

**FIGURE 1:**
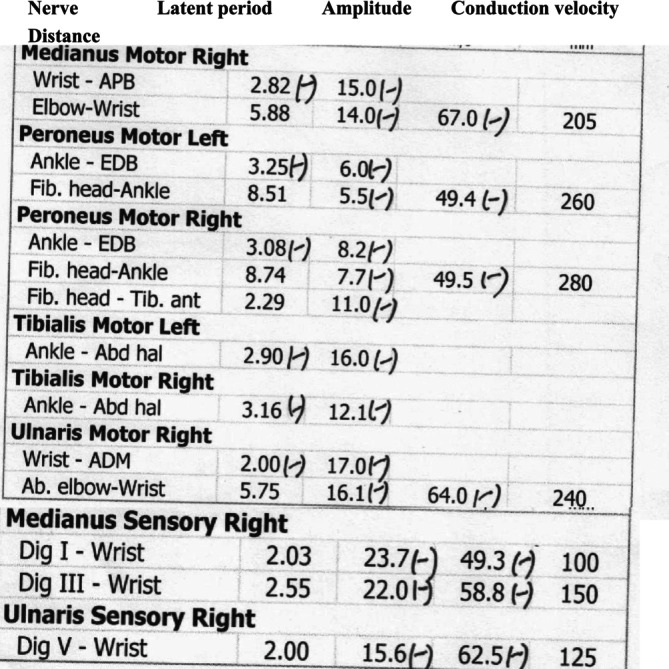
Motor and sensory nerve electromyography showed varying degrees of motor and sensory nerve damage.

**FIGURE 2:**
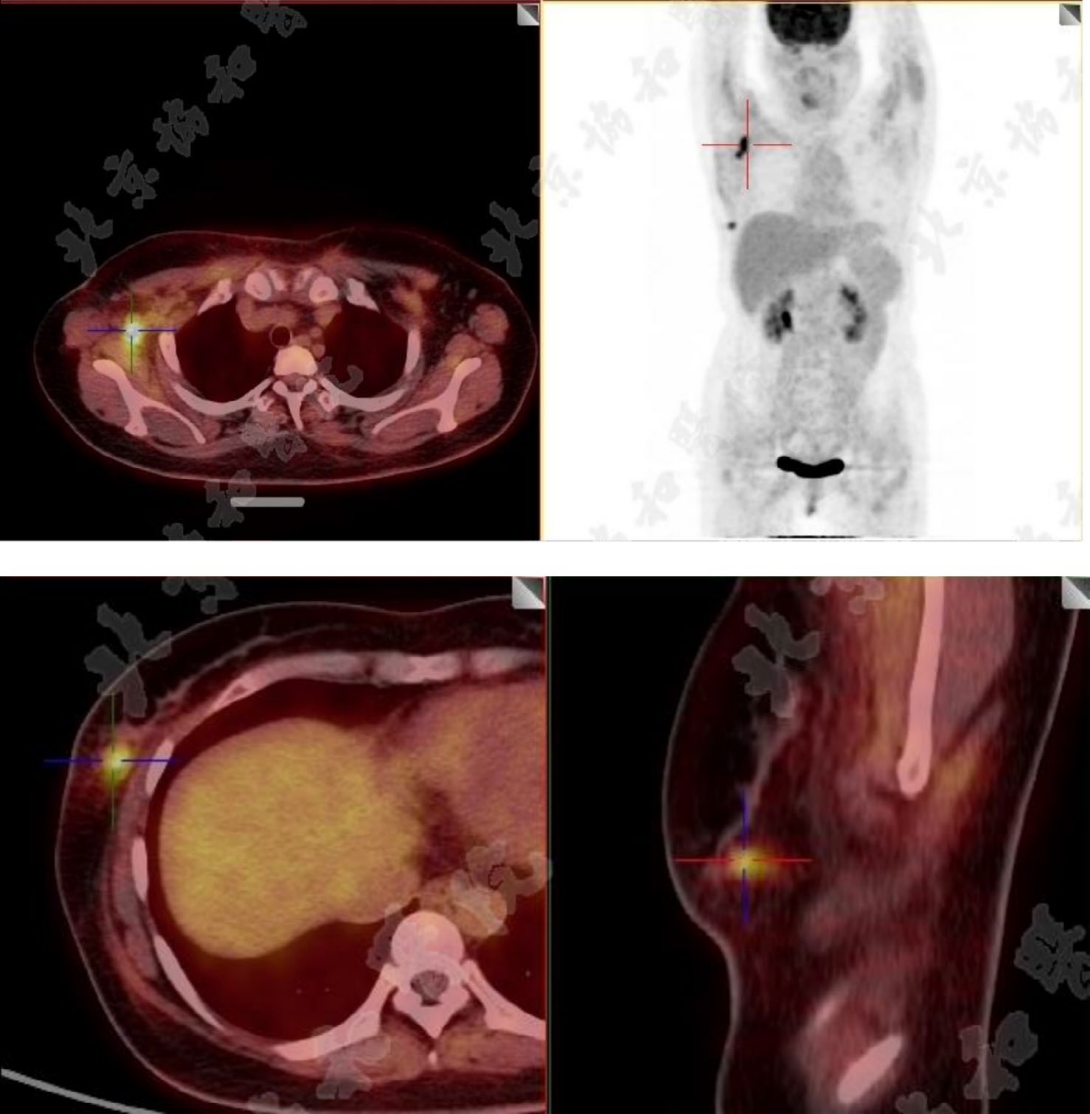
PET‐CT showed right‐sided breast cancer and multiple lymph node metastases in the armpit.

She was admitted to our hospital, consulting the emergency doctor first on August 30, 2023. Besides skin cutaneous manifestations, proximal muscle strength was checked with score 4−/5, without changes in distal muscles (Figure 1). There was no significant difference in skin manifestations before. Electromyography showed varying degrees of damage to both motor and sensory nerves. Laboratory examinations showed that CK (creatine kinase) 1010 U/L, CK‐MB (creatine kinase isoenzymes) 9.9 μ/L, Myo (myoglobin) 252 μ/L, ANA(+), Anti‐TIF1‐γ(+), Neutrophils 81%, and SaO_2_ 90%. Other autoimmune‐related indicators and tumor‐related signs were negative. Aspartate transaminase and lactate dehydrogenase were elevated. Potassium, sodium, and calcium ions were generally normal. A CT scan showed mild interstitial lung disease, and a PET‐CT showed nodules with elevated metabolism in the outer lower quadrant of the right mammary (Figure 2), which was consistent with malignant lesions; multiple lymph node metastasis was screened in the right axillary; there was no distal metastasis. The Emergency physician had a high suspicion of tumor‐associated DM and consulted with a dermatologist, rheumatologist, and breast surgeon. The results of skin and pectoral muscle biopsies suggested DM. Tumor‐related DM was diagnosed by the breast surgeon and rheumatologist. For triple‐negative breast cancer, neoadjuvant chemotherapy is recommended. However, due to the severity of DM‐related manifestations, especially the muscle damage that caused the patient to have dyspnea, low oxygen saturation that was conducive to general anesthesia, and severe skin lesions that affected the surgical wound healing, and the patient had no need for breast conservation, it was suggested that the patient be admitted to the Rheumatology department for the treatment of DM first.

## 4 Conclusions

After 1 month of active treatment by the rheumatologist, mainly prednisone, with a maximum dose was 60 mg/d, methotrexate was also added. The initial dose of MTX was 5 ~ 7.5 mg/week, increasing by 2.5 ~ 5 mg per week, and the target dose was 10 ~ 20 mg/week. Immunoglobulin was injected for consecutive 5 days. CK, CKMB, and Myo indicators returned to normal gradually; skin lesions, dyspnea, blood oxygen saturation, choking cough, and muscle strength 5−/5 significantly improved. Through the preoperative evaluation of anesthesiology, the patient was transferred to the Department of Breast Surgery for modified radical mastectomy, and the pathological results showed invasive breast carcinoma (non‐special type, medium‐low differentiation, size 3 × 1.5 × 1 cm), multiple intravascular tumor emboli, lymph node metastatic carcinoma (right axillary 7/12) and immunohistochemical results: Estrogen receptor (ER/−), progesterone receptor (PR/−), human epidermal growth factor receptor 2 (Her2/−), Ki‐67 (80%). Postoperatively, chemotherapy with epirubicin hydrochloride 150 mg and cyclophosphamide 900 mg was first administered every 2 weeks for 4 courses, followed by docetaxel 150 mg every 3 weeks for 4 courses. Then capecitabine 1500 mg was taken orally twice daily for two consecutive weeks and stopped for 1 week. This process must be completed for at least 6 cycles, combined with radiotherapy for at least 20 times. The patient is currently in the process of chemotherapy.

## 5 Discussion

### 5.1 DM and Malignancy

The incidence of DM is lower than that of tumors, but DM can increase the risk of malignant tumors, and the incidence of DM complicated by tumors varies greatly [10]. Patients with DM are at much higher risk of developing malignancy than the normal population. Malignant tumors are one of the main causes of death in patients with DM [11]. Therefore, early awareness of the possible existence of potential malignant tumors and timely detection are of great significance for the quality of life and prognosis of patients with DM. DM can occur before malignancy, after treatment of malignancy, or both at the same time, and DM‐related symptoms do not improve significantly after corticosteroid treatment, but after malignant tumor resection surgery or related anti‐tumor treatments such as chemotherapy and radiotherapy, these symptoms can improve significantly [12]. The mechanism of DM associated with malignant tumors may be closely related to genetics, immune dysfunction, viral infection, and immune cross‐reactivity, but the specific mechanism is not clear [13].

Breast cancer is the most common malignant tumor in women. The incidence is about 59.0/100,000 in Chinese women [14]. DM is always coupled with breast cancer, which has been supported by a large number of retrospective studies [15]. People with DM are nearly six times more likely to develop malignancies than the general population [16]. The incidence in patients with malignant tumors is only second to that of uterine malignancies [17]. Characteristics of patients with DM and breast cancer: the age of onset is mostly over 40 years which is consistent with the fact that age > 40 years is a risk factor for DM complicated with malignancy. The median time between the diagnosis of breast cancer and the onset of DM symptoms is 1 month, with the highest risk of developing breast cancer, especially in the first 2 years after the initial diagnosis of DM [18]. The most common type of breast cancer in patients with DM and breast cancer is invasive ductal carcinoma [16]. Most breast cancer patients with DM have a late tumor stage (stage III or IV). There was no significant difference in positive rates of ER, PR, and HER‐2 in breast cancer patients with DM [19]. However, in our case report, these patients were characterized by triple‐negative breast cancer, though the result is not based on statistical analysis.

### 5.2 Treating Experience for DM and Breast Carcinoma

At present, there is no guideline for the management of DM complicated with breast cancer, and there is no prospective data on the treatment regimen, and the specific treatment regimen needs to be individualized according to the patient's situation. Most of the case reports suggest that the treatment of DM complicated with breast cancer is still based on the principle of breast cancer treatment. Neoadjuvant chemotherapy is the primary systemic treatment for TNBC, which improves pathological remission, improves surgical success, reduces the extent of surgery, and allows assessment of treatment response. The treatment of DM is mainly based on systemic corticosteroids. It is critical to determine the timing of treatment for breast cancer and DM.

However, there are some conflicts between treating DM‐related manifestations first and treating breast cancer first. Some scholars reported that severe muscle symptoms should be controlled with steroids before radical excision of breast cancer; then mastectomy and postoperative adjuvant therapy were started after remission of DM‐related symptoms [17, 20]. TNBC, which is a special subtype of breast cancer, has been demonstrated to have a significantly higher risk of recurrence and death compared with luminal subtypes. Therefore, some scholars opted for surgery first after significant tumor shrinkage with neoadjuvant chemotherapy and postoperative anti‐DM treatment with steroid drugs and methotrexate. They think treatments for DM are even not necessary because these symptoms can be relieved. Moreover, if only skin rash symptoms are found without muscle symptoms, the cutaneous manifestations can be relieved by neoadjuvant therapy combined with or without DM‐related treatment before surgery to promote wound healing and prevent postoperative infection [8, 12, 21]; some scholars reported executing both neoadjuvant and DM‐related treatment at the same time, the patient's DM symptoms had largely recovered, mastectomy was then performed, and the patient recovered well. Initiating oral corticosteroid therapy and neoadjuvant chemotherapy concurrently, after significant tumor shrinkage, mastectomy can be conducted [22].

In our perspective, we need to adopt different treatment strategies for different patients: among the 3 patients in this group, there are serious skin problems, muscle problems, intermittent dyspnea, and interstitial diseases of the lungs. If these DM‐related symptoms are not well controlled before surgery, the risk of anesthesia during surgery is high. Hence, for lung problems and dyspnea, anesthesiology consultation is required before surgery, and if necessary, respiratory consultation is required to evaluate whether the patient can tolerate general anesthesia. Of course, some scholars believe that it takes a long time to control the symptoms of DM, which affects the treatment of tumors, and the removal of the tumor under intravenous anesthesia can be carried out. Khoo et al. reported a 63‐year‐old female patient with DM and breast cancer [23]. Because the patient was in poor general condition due to severe muscle weakness and dysphagia, breast cancer resection under local anesthesia by the method of swelling anesthesia was carried out, and the postoperative pathology showed negative resection margins. The tumor was completely resected, and the patient's muscle strength improved more obviously than before 5 months after surgery. However, it is recognized as very aggressive. At the same time, acute skin problems may lead to poor postoperative wound healing, and patients may also take large doses of corticosteroids, which may also lead to poor wound healing. These patients are also unable to tolerate neoadjuvant chemotherapy for breast cancer. Therefore, in this paper, we recommend that the symptoms related to DM be controlled first, and then breast cancer resection should be carried out. If the patient does not have serious skin and respiratory problems, neoadjuvant chemotherapy and DM can be treated at the same time, and the breast mass can be reduced and surgery for breast cancer can be performed as soon as possible. Therefore, for patients with DM combined with breast cancer, different patients need to adopt different strategies, and there is no one standard strategy. Clinicians from multiple departments need to jointly evaluate which treatment must be put first, including breast surgery, dermatology, Rheumatology, anesthesiology, etc.

**FIGURE 3:**
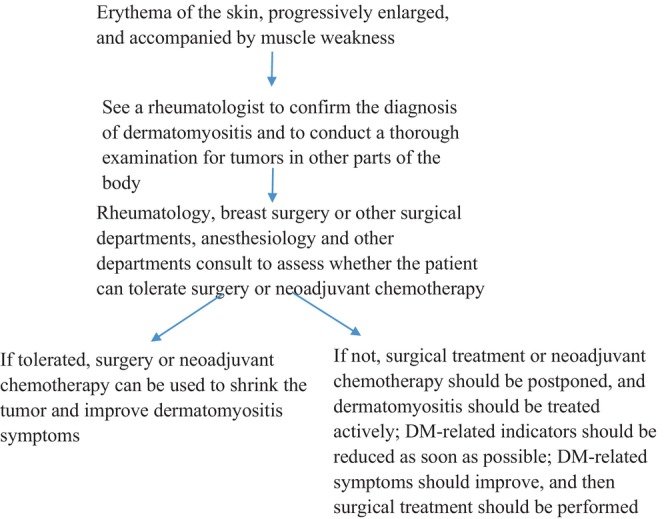
Treatment for DM and Breast cancer guideline.

Treatment guideline is listed below in Figure 3.

### 5.3 Anti‐TIF1‐γ Antibody and TNBC

The TIF1‐γ antibody was first reported as a member of the TIF‐1 gene family in 1998 [24]. The anti‐TIF1‐γ antibody is identified in about 20% of DM patients [25]. It plays a role in transcriptional elongation, cell differentiation, embryonic development, DNA repair, and mitosis [26]. Some scholars have speculated that mutations in the TIF1‐γ gene can elicit a specific anti‐tumor immune response, which in turn forms cross‐immunity with target organs such as muscle and skin [27]. Moreover, it may be associated with different kinds of cancer via certain signaling pathways. Tumor cells express high levels of MSAs, such as anti‐TIF1 antibodies, which are clearly associated with potentially malignant tumors [28]. It is hypothesized that anti‐TIF1 antibodies are produced in anti‐tumor immune responses. Some reported that facial DM and the shawl sign were more common in patients with TIF1‐γ [29]. The shawl sign (V‐neck sign) was more commonly seen than other symptoms. In a case of DM complicated with breast cancer reported by Tina Hu et al. [30], DM was confirmed by skin biopsy for the presentation of sign V rash, negative for antinuclear antibodies, double‐stranded DNA, and rheumatoid factor, with normal complement levels, and positive for TIF1‐γ antibodies. Due to the strong association between TIF1‐γ antibodies and malignancy, the patient was screened for malignancy, and the initial mammogram showed no obvious abnormalities. A few months later, a unilateral breast mass was palpated, and the diagnosis of invasive breast cancer was confirmed. There is a meta‐analysis showing that the association between anti‐TIF1‐γ antibody and malignant tumor complications had a sensitivity of 78%, a positive predictive value of 58%, and a negative predictive value of 95% [31]. Another interesting study showed that 72% of anti‐TIF1‐γ antibody‐positive patients over 40 years old had malignant tumors [32]. Hence, it is important to screen for malignant tumors within 1–2 years after the onset of DM in patients over 40 years old.

As a key diagnostic indicator for DM, the coexistence of anti‐TIF1‐γ positive and triple‐negative breast cancer appeared in 4 cases. No research has been found on the relationship between them after query on Pubmed. It is unclear whether there is a positive result that a higher probability of triple‐negative breast cancer can be spotted in the patient with anti‐TIF1‐γ positive or whether the coexistence in this group is only a coincidence. Triple‐negative breast cancer accounts for 10.0% ~ 20.8% of all pathological types of breast cancer. It has special biological behavior and clinicopathological characteristics, and its prognosis is worse than that of other types. At present, there is no specific treatment guidelines. As one of the largest breast surgery centers in the country, we will continue to collect information on patients with anti‐TIF1‐γ positive and triple‐negative breast cancer, hoping to find out whether there is a certain relationship between them, which will help us evaluate the effect of chemotherapy and prognosis of triple‐negative positive breast cancer patients after surgery.

## 6 Conclusion

In this case report about anti‐TIF1‐γ positive and triple‐negative breast carcinoma, we would like to point out two key points: for treating DM or triple‐negative breast cancer first, different strategies need to be specified according to different situations. The severity of DM needs to be assessed before surgery, and if the respiratory muscle symptoms of DM are not eligible for surgical treatment, it is recommended to treat the symptoms of DM first and observe whether neoadjuvant chemotherapy can be used at the same time to reduce the tumor volume and create the possibility of radical breast cancer surgery or breast‐conserving surgery; very few published reports (just two articles reporting only one case respectively) have shown that anti‐TIF1‐γ antibody‐positive and triple‐negative breast cancer occur at the same time. Therefore, close correlation between anti‐TIF1‐γ antibody‐positive and triple‐negative breast cancer should draw our attention.

## Author Contributions

**Fan Zhang:** conceptualization, data curation. **Hailing Guo:** resources, validation. **Jia Ma:** formal analysis, investigation.

## Ethics Statement

A written informed consent was obtained from the patient to publish this report in accordance with the journal's patient consent policy.

## Conflicts of Interest

The authors declare no conflicts of interest.

## Data Availability

All the data and materials are from the Medical Record Department of our hospital.
